# Radiation-Induced miRNAs Changes and cf mtDNA Level in Trauma Surgeons: Epigenetic and Molecular Biomarkers of X-ray Exposure

**DOI:** 10.3390/ijms25158446

**Published:** 2024-08-02

**Authors:** Assiya Kussainova, Akmaral Aripova, Milana Ibragimova, Rakhmetkazhi Bersimbaev, Olga Bulgakova

**Affiliations:** Department of General Biology and Genomics, Institute of Cell Biology and Biotechnology, L.N. Gumilyov Eurasian National University, Astana 010008, Kazakhstan; assya.kussainova@gmail.com (A.K.); aripova001@gmail.com (A.A.); milanaibragimova2602@yandex.ru (M.I.); ribers@mail.ru (R.B.)

**Keywords:** miRNA, cf mtDNA, cytokines, X-ray

## Abstract

Exposure to ionizing radiation can result in the development of a number of diseases, including cancer, cataracts and neurodegenerative pathologies. Certain occupational groups are exposed to both natural and artificial sources of radiation as a consequence of their professional activities. The development of non-invasive biomarkers to assess the risk of exposure to ionizing radiation for these groups is of great importance. In this context, our objective was to identify epigenetic and molecular biomarkers that could be used to monitor exposure to ionizing radiation. The impact of X-ray exposure on the miRNAs profile and the level of cf mtDNA were evaluated using the RT-PCR method. The levels of pro-inflammatory cytokines in their blood were quantified using the ELISA method. A significant decrease in miR-19a-3p, miR-125b-5p and significant increase in miR-29a-3p was observed in the blood plasma of individuals exposed to X-ray. High levels of pro-inflammatory cytokines and cf mtDNA were also detected. In silico identification of potential targets of these miRNAs was conducted using MIENTURNET. VDAC1 and ALOX5 were identified as possible targets. Our study identified promising biomarkers such as miRNAs and cf mtDNA that showed a dose-dependent effect of X-ray exposure.

## 1. Introduction

People are regularly exposed to a range of ionizing radiation. Individuals engaged in work involving radiation sources are also exposed as part of their professional activities. This category of workers includes trauma surgeons, who perform various manipulations on a regular basis in order to treat injuries and monitor the condition of patients.

Fluoroscopy represents one of the principal methods for the noninvasive visualization of injuries. Nevertheless, despite the numerous advantages of X-ray machines, there is a significant risk of radiation exposure for the surgical team. X-ray belongs to the class of ionizing radiation. Its use is not limited to tissue visualization; it is actively used in radiation therapy for oncological diseases [1].

The negative effects of ionizing radiation on the human body are well documented, with the potential to lead to the development of a number of diseases, including cataracts [2] and cancer [3]. It is noted that small doses of radiation can have a cumulative effect on the body [4].

The effect of radiation is associated with two types of risk: stochastic, which refers to the consequences of random mutations, and deterministic, which refers to the probable effects of exposure. The use of fluoroscopic methods in medical practice is associated with stochastic risk [5]. The International Commission on Radiological Protection (ICRP) has established a reference level for occupational radiation exposure of 1 mSv per year [6].

The extent to which trauma surgeons are exposed to X-ray depends on a number of factors: the type and length of exposure, the position of the C-arm, the position of the surgeon and the radiation protection equipment used [7].

In a study conducted by Hurley et al., the efficacy of radiation-protection equipment was evaluated. It was determined that the use of such equipment during procedures involving fluoroscopy significantly reduces the radiation dose to the body [8]. Nevertheless, regions of the body not safeguarded by lead plates remain susceptible to radiation exposure.

Tissues damaged by radiation frequently exhibit pronounced signs of inflammation. It is well established that the expression of pro-inflammatory cytokines (e.g., TNF-α, IL-1, IL-6, IL-8, IFN, G-CSF, VEGF and EGFR) occurs within hours of exogenous exposure. The pro-inflammatory phase persists throughout the entire period of radiation exposure and can develop into chronic inflammation [9]. Pro-inflammatory interleukin-6 (IL-6) is synthesized at the initial stage of inflammation [10] and contributes to the progression of the inflammatory response [11]. Multiple studies have reported that IL-6 is an inflammatory marker and shows good potential for use in the diagnosis of inflammatory diseases [12,13]. Tumor necrosis factor alpha (TNF-α) is a key mediator of the inflammatory response. Elevated levels of TNF-α have been observed in both acute and chronic inflammatory conditions [14].

Dosimeters are employed to monitor the dose of radiation exposure. Nevertheless, the use of measuring instruments enables the total radiation dose received by a surgeon during their work to be quantified. This information is of significant importance, as it allows for the calculation of risks and the determination of preventive measures. However, the data obtained from the dosimeter cannot report on the changes that have already occurred, caused by X-rays at the molecular and cellular levels. Consequently, the search for biological markers of radiation exposure is an urgent task.

MicroRNAs (miRNAs) are short non-coding RNA molecules, 19–25 nucleotides in length, which function as epigenetic regulators of gene expression. A considerable body of evidence indicates that miRNAs have the potential to serve as promising biomarkers for a range of pathological conditions. Furthermore, their profile is susceptible to external factors [15]. The detection of cell-free miRNAs in blood plasma renders them the most accessible for analysis. Previous studies have demonstrated a correlation between ionizing radiation exposure and miRNAs [16,17]. A study investigating the miRNAs profile of interventional cardiologists who were occupationally exposed to radiation revealed a reduction in the expression of miR-134 and miR-2392 in the brain [18]. Similarly, another study examined the expression of candidate genes in workers at the Mayak nuclear facility and found altered mRNA and miRNA gene expression [19]. Consequently, the detection of miRNA signatures when trauma surgeons are exposed to X-rays represents a priority area of research with considerable potential.

Ionizing radiation can take an indirect path by increasing the level of reactive oxygen species (ROS). In normal conditions, ROS are secondary messengers participating in various physiological processes of the organism [20]. But when the level of ROS increases, oxidative stress occurs, which leads to a disturbance in the integrity of nucleic acids [21]. According to the study conducted by Kim et al., ionizing radiation indirectly enhances mitochondrial biogenesis and the expression of mitochondrial genes through its destructive effects. Most likely, the reason is that cells, in an attempt to compensate for radiation-induced mitochondrial dysfunction and increase their energy reserves, begin to increase the number of copies of mitochondrial DNA [22]. Mitochondrial DNA (mtDNA) is released into the bloodstream in both normal and pathological conditions through various physiological and pathological mechanisms, for example, during apoptotic cell death [23]. Plasma cell-free mitochondrial DNA (cf mtDNA) levels increase in populations exposed to radon [24]. The number of studies on changes in the level of circulating cf mtDNA in response to various triggers continues to grow. Regarding the question of the impact of ionizing radiation on mtDNA, it is believed that mtDNA is more susceptible to radiation than nuclear DNA, as mtDNA lacks histones and therefore is not protected by them, and it lacks an efficient repair system. In addition, mtDNA is located in close proximity to the electron transport chain (ETC), which makes it more sensitive to ROS [21,25]. In summary, it is evident that cf mtDNA could be a promising biomarker for assessing radiation exposure.

The purpose of this study was to investigate the potential of using targeted miRNAs and cf mtDNA as biomarkers of X-ray exposure. 

## 2. Results

### 2.1. Characteristics of the Experiment Participants

The study involved 86 people, of whom 30 were trauma surgeons and 56 people were assigned to the control group. Comparative characteristics of the study participants are presented in Table 1. 

### 2.2. Individual Annual Effective Doses for Trauma Surgeons

The values of individual annual effective doses (AED) are presented in Table 2. The average annual effective dose for trauma surgeons was 0.6 mSv.

### 2.3. The Level of miRNA Expression in the Blood Plasma of Individuals Exposed to X-rays

We studied the relative expression level of the following miRNAs: miR-19a-3p, miR-29a-3p, miR-125b-5p, miR-142-5p, miR-144-5p, miR-150-5p, miR-181a-3p and miR-320c in the blood plasma of individuals exposed and not exposed to X-rays.

**Figure 1:**
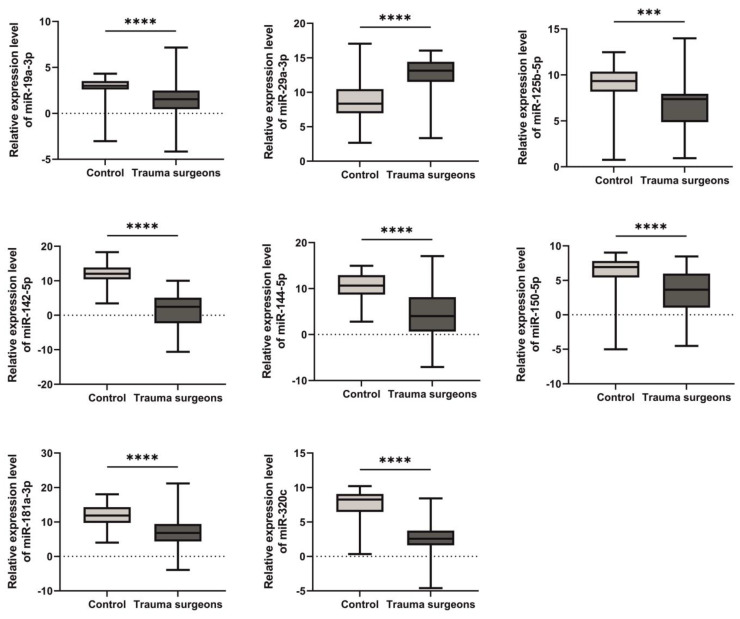
Graphical summary of the relative expression of miRNA in the blood plasma of individuals exposed to X-rays. The expression level of miR-19a-3p was 1.8-fold lower in the plasma of individuals exposed to X-rays compared to those who were not exposed. The expression level of miR-29a-3p was 1.4-fold higher in the blood plasma of trauma surgeons compared to the control group. miR-125b-5p showed a 1.26-fold decrease in expression level in the blood plasma of individuals exposed to X-rays compared to the control group. The level of miR-142-5p in the blood plasma of trauma surgeons was 10.4-times lower compared to the control group. The expression level of miR-144-5p was 2.2-fold lower in the plasma of individuals who received a dose of X-rays compared to individuals who were not exposed. The expression level of miR-150-5p in the blood plasma of trauma surgeons was 1.6-times lower compared to the control group. The expression level of miR-181a-3p was 1.8-fold lower in the group of individuals who were exposed to X-rays compared to those who were not exposed. miR-320c showed a 3.5-fold decrease in expression in the group of trauma surgeons compared to the control group. *** *p* ≤ 0.001; **** *p* ≤ 0.0001.

A significant decrease in miR-19a-3p, miR-125b-5p, miR-142-5p, miR-144-5p, miR-150-5p, miR-181a-3p and miR-320c was observed in the blood plasma of individuals exposed to X-rays (Figure 1).

The expression level of miR-29a-3p was increased in the blood plasma of individuals exposed to X-rays (Figure 1).

### 2.4. Analysis of the Correlation between the miRNA Profile and Annual Effective Dose of Trauma Surgeons

**Figure 2:**
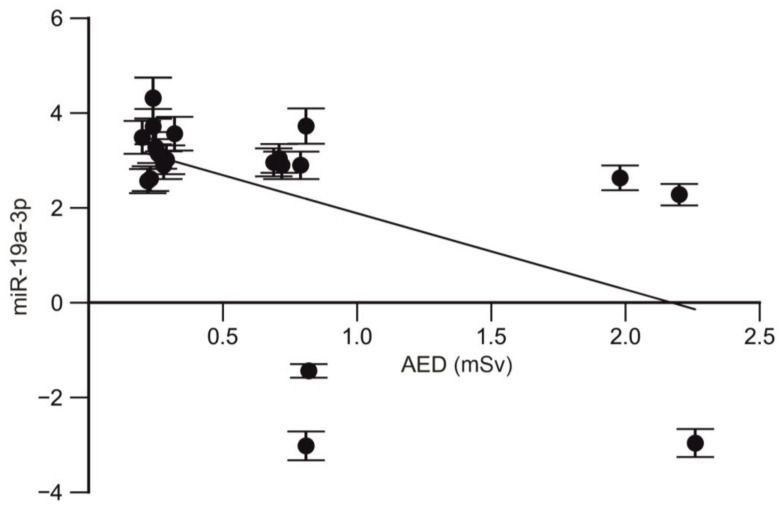
Correlation between the expression level of miR-19a-3p and AED.

A strong correlation was also found between the expression level of miR-19a-3p and the annual effective dose in trauma surgeons (r = −0.52; *p* = 0.015; CI95% −0.7839 to −0.1037; Figure 2).

**Figure 3:**
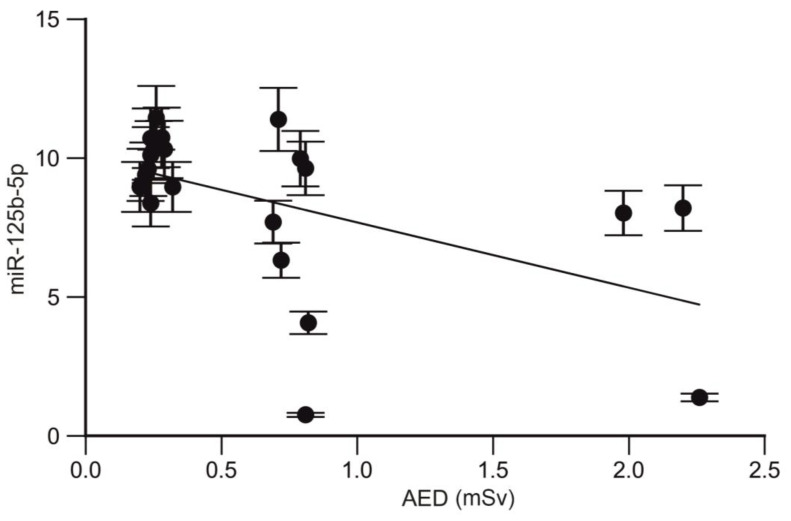
Correlation between the expression level of miR-125b-5p and AED.

A strong correlation was also found between miR-125b-5p and the annual effective dose in trauma surgeons (r = −0.49; *p* = 0.022; CI95% −0.7695 to −0.06774; Figure 3).

### 2.5. Analysis of the Correlation between the miRNA Profile and Length of Employment of Trauma Surgeons

**Figure 4:**
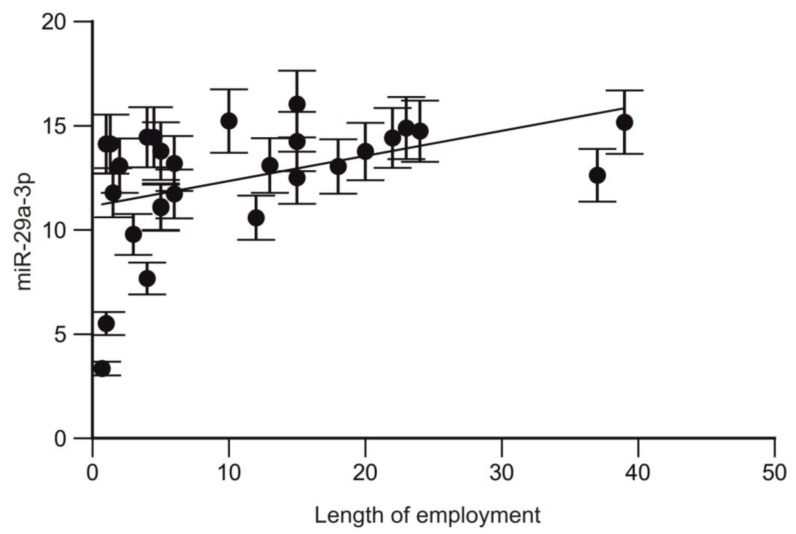
Correlation between the expression level of miR-29a-3p and the length of employment.

A positive correlation was also found between miR-29a-3p and the length of employment in trauma surgeons (r = 0.47; *p* = 0.011; CI95% 0.1100 to 0.7248; Figure 4).

### 2.6. The cf mt Copy Numbers in the Blood Plasma of Individuals Exposed to X-ray

The median cf mtDNA in the control group, not exposed to occupational X-ray radiation, was 1.21 × 10^5^ copies/mL. In the trauma surgeons, the median was 5.28 × 10^5^ copies/mL (Table 3).

**Figure 5:**
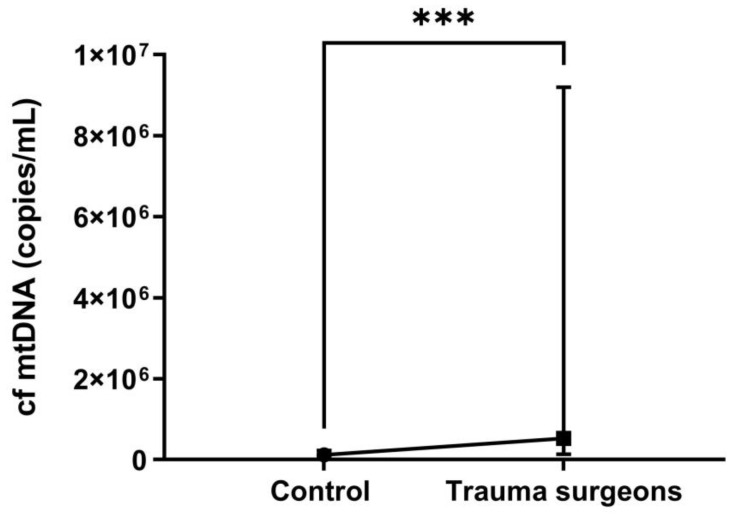
The copies number of cf mtDNA in the blood plasma of control and trauma surgeons occupationally exposed to X-rays. *** *p* ≤ 0.001.

The obtained results demonstrate that the level of cf mtDNA in the blood samples of trauma surgeons is more than four times higher than in the control group (*p* = 0.0005) (Figure 5).

**Figure 6:**
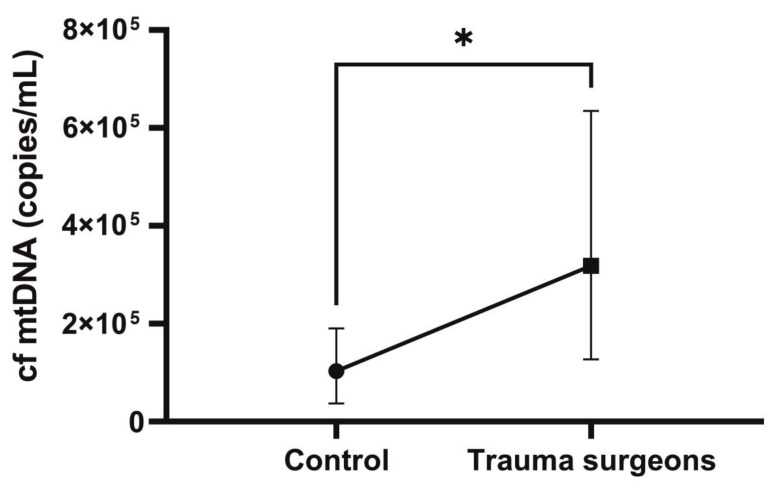
The copy number of cf mtDNA in the blood plasma of control and nonsmoker trauma surgeons. * *p* < 0.05.

The level of cf mtDNA copies was nearly three times higher in non-smoking trauma surgeons compared to the control group (*p* = 0.01) (Figure 6).

**Figure 7:**
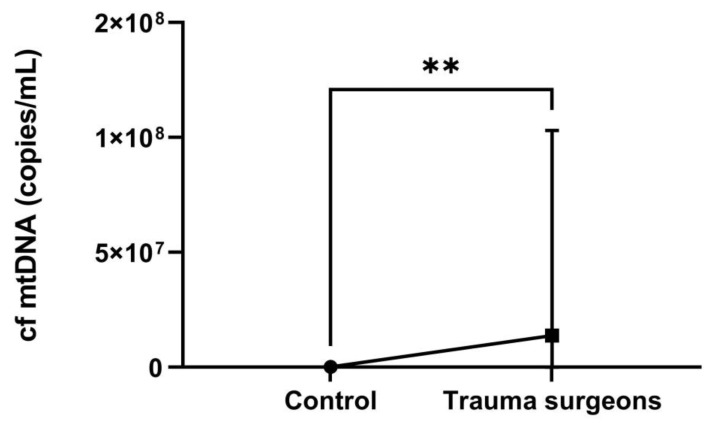
The copy number of cf mtDNA in the blood plasma of smokers in the control group and trauma surgeons that smoke. ** *p* < 0.01.

It is established that smoking can also impact the level of cf mtDNA [26]. We also compared this indicator among smokers in both groups. The difference in copy numbers of the cf mtDNA was significantly higher by 95 times in trauma surgeons who smoked (*p* = 0.003) (Figure 7). This suggests a synergistic effect of smoking and X-rays on the organism.

### 2.7. Analysis of the Correlation between the Level of cf mtDNA and Annual Effective Dose of Trauma Surgeons

**Figure 8:**
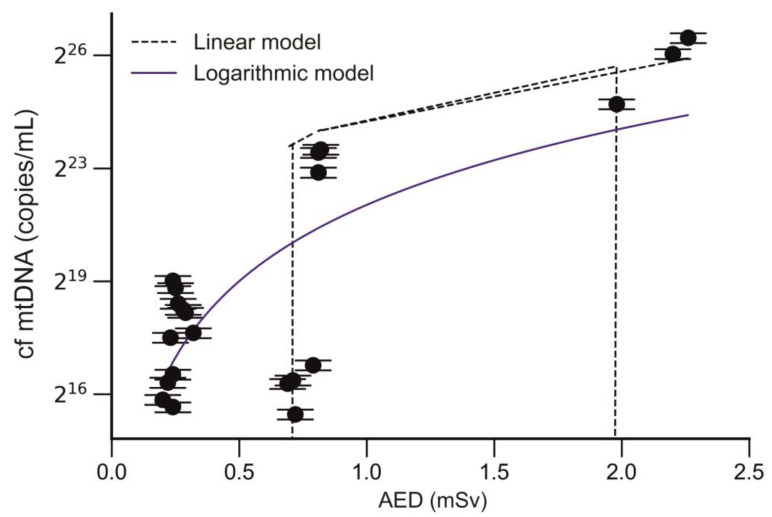
Correlation of cf mtDNA level and AED.

A strong correlation was also found between the cf mtDNA level and the annual effective dose in trauma surgeons (r = 0.6; *p* < 0.002, CI95% 0.2501 to 0.8357; Figure 8).

### 2.8. Analysis of the Impact of Age, Gender and Smoking on Changes in miRNAs Profile and cf mtDNA Level

The relationship between the miRNAs profile and cf mtDNA level and age, gender and smoking is also shown in Table 4. No effect of age, sex and smoking on miRNA profile changes was observed. There was also no effect of sex and age on cf mtDNA levels.

### 2.9. Prognostic Potential of miRNAs and cf mtDNA in Individuals Exposed to X-rays

ROC curves showed a high sensitivity and specificity for miRNAs and cf mtDNA and suggested the possibility of using them as biomarkers of exposure to X-rays.

**Figure 9:**
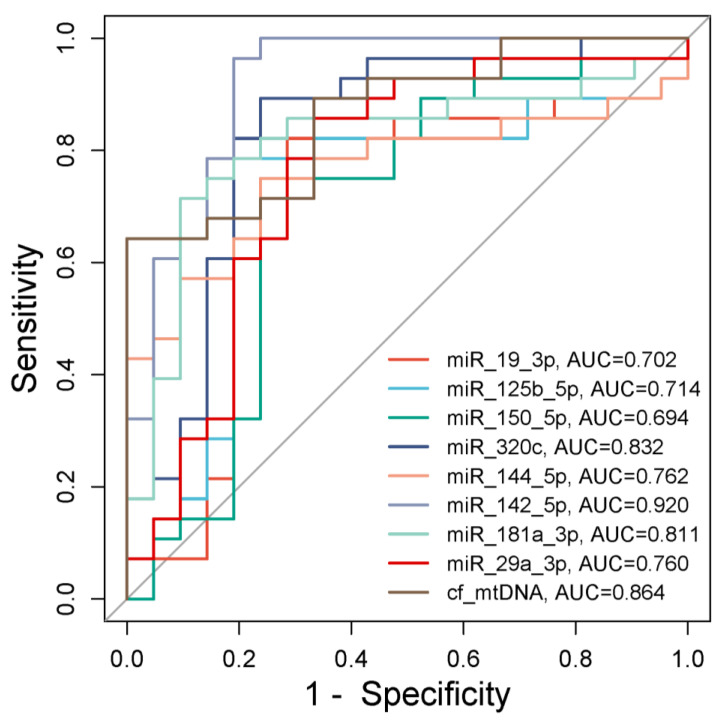
ROC curve for miRNAs and cf mtDNA based on the RT-qPCR data.

Accordingly, miRNAs and cf mtDNA may be considered as valuable biomarkers of X-ray exposure (Table 5, Figure 9).

### 2.10. Levels of TNF-α, IL-6 and IL-4 in the Blood Serum of Individuals Exposed to X-rays

The spectrum of pro- and anti-inflammatory cytokines in the blood serum of trauma surgeons who were exposed to X-rays was studied.

**Figure 10:**
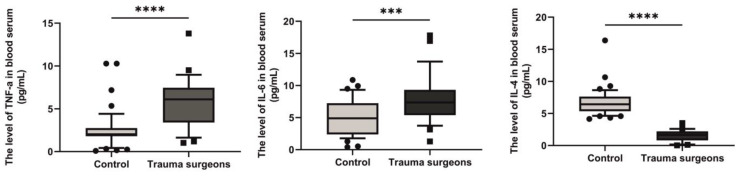
Levels of pro- and anti-inflammatory cytokines in the blood serum of individuals non-exposed and exposed to X-rays. *** *p* < 0.001; **** *p* < 0.0001.

The median pro-inflammatory TNF-α in individuals exposed and not exposed to X-rays was 6.103 pg/mL and 1.916 pg/mL, respectively. The level of TNF-α in individuals exposed to X-rays was 3.2 times higher than in the control group (*p* < 0.0001) (Figure 10).

The median pro-inflammatory IL-6 in individuals exposed and not exposed to X-rays was 7.356 pg/mL and 4.9 pg/mL, respectively. The level of IL-6 in individuals exposed to X-rays was 1.5 times higher than in the control group (*p* < 0.0001) (Figure 10).

The median anti-inflammatory IL-4 in individuals exposed and not exposed to X-rays was 1.655 pg/mL and 6.42 pg/mL, respectively. The level of IL-4 in individuals exposed to X-rays was 3.9 times lower than in the control group (*p* < 0.0001) (Figure 10).

### 2.11. Analysis of the Correlation between the Spectrum of Pro- and Anti-Inflammatory Cytokines and Length of Employment of Trauma Surgeons

**Figure 11:**
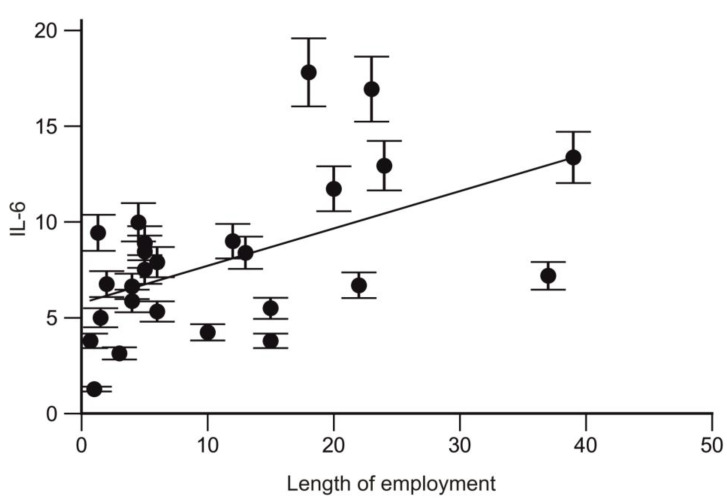
Correlation between the expression level of IL-6 and the length of employment.

A positive correlation was also found between IL-6 and the length of employment in trauma surgeons (r = 0.51; *p* = 0.006; CI95% 0.1496 to 0.7583; Figure 11).

### 2.12. Search for a Correlation between the Profile of miRNAs and Pro- and Anti-Inflammatory Cytokines

**Figure 12:**
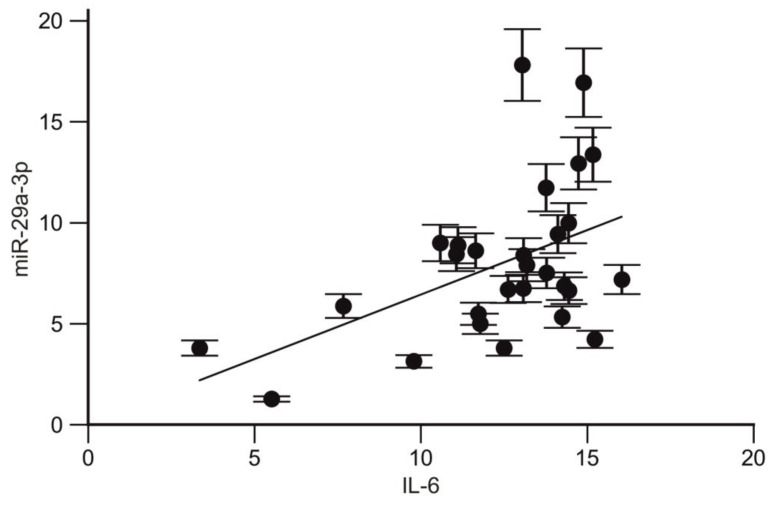
Correlation between the expression levels of miR-29a-3p and IL-6.

We found that the expression levels of miR-29a-3p and IL-6 were significantly correlated when exposed to X-rays (r = 0.394; *p*= 0.03; CI95% 0.01766 to 0.6777; Figure 12).

### 2.13. Target Enrichment Analysis Results

**Figure 13:**
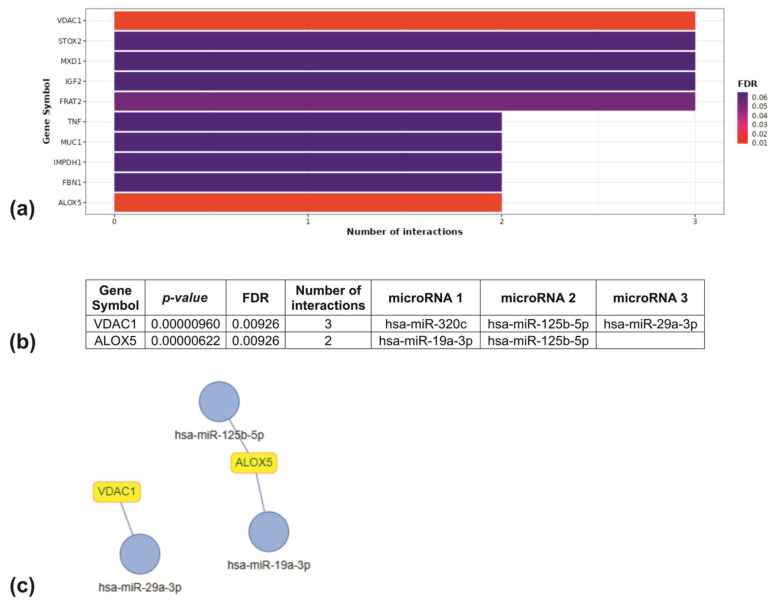
Outputs of the MIENTURNET web tool. (**a**) Number of interactions between miRNAs and target genes predicted by miRTarBase using the MIENTURNET algorithm. (**b**) miRNA–target enrichment analysis result. (**c**) miRNA–target interaction network constructed using MIENTURNET. Blue dots represent miRNAs, yellow blocks represent miRNA targets.

miRNA–target enrichment analysis identified two target genes for four target miRNAs. miR-19a-3p and miR-125b-5p interact with arachidonate 5-lipoxygenase (ALOX5). Voltage-dependent anion-selective channel 1 (VDAC1) is a target of miR-320c, miR-125b-5p and miR-29a-3p (Figure 13a).

For network miRNA/genes analysis, miRTarBase constructed the following interaction networks. The interaction of miRNAs with targeted genes is as follows: miR-29a-3p interacts with VDAC1, and miR-19a-3p and miR-125b-5p interact with ALOX5 (Figure 13b,c).

## 3. Discussion

The performance of surgical procedures in the field of traumatology necessitates the utilization of fluoroscopic visualization techniques to facilitate the operative field. This procedure results in the exposure of patients and trauma surgeons to varying doses of X-rays. Trauma surgeons perform such manipulations on a regular basis, thereby exposing themselves to chronic exposure to X-rays. In order to ensure radiation safety, many countries around the world have established hygienic standards. In Brazil, this indicator is set at a limit of 20 mSv per year [27]; in Turkey, it is no more than 6 mSv per year [28]. In the Republic of Kazakhstan, the effective dose is determined to be 20 mSv per year on average for any five consecutive years of operation [29]. There is an association between exposure to ionizing radiation, including X-rays, and the development of a number of diseases, including cataracts [30] and oncology [31]. It is well established that such radiation can induce thyroid cancer [32], melanoma [33], glioma [34], leukemia [35], skin cancer [36] and breast cancer [37]. Despite the availability of a plethora of protective measures for medical personnel, including lead aprons, protective collars and glasses, teams engaged in work with sources of ionizing radiation remain at risk of developing diseases associated with such exposure. It has been demonstrated that lead aprons are capable of blocking in excess of 90% of incident X-rays. miRNA molecules respond to external factors, including exposure to ionizing radiation [38]. An increasing number of studies in which miRNA molecules are studied as biomarkers have shown that miRNAs can be used for the diagnosis, prognosis and monitoring of various conditions and diseases. For example, many studies have shown that miRNA molecules are associated with radiation-induced cellular senescence [39]. The profile of freely circulating miRNAs is determined in blood plasma, making them easily accessible biomarkers for analysis.

There are many studies that examine the role of specific miRNAs as biomarkers for radiation exposure. Templin et al. identified changes in the profile of 31 miRNAs in the blood of mice that were exposed to high and low doses of radiation. Of these, miR-150 changed its expression level after irradiation with different radiation sources and exposure time [40]. Similar data were obtained by Jacob et al., who demonstrated a dose-dependent reduction in miR-150 levels in blood plasma following exposure to ionizing radiation [41]. Song et al. propose six miRNAs that may be potential biomarkers for assessing radiation exposure: miR-145, miR-663, miR-1307-3p, miR-6090, miR-6727-5p and miR-7541 [17]. According to a study by Halimi et al., serum miR-34a can be used as an indicator of radiation exposure [42]. Zhang et al. identified 56 exosomal miRNAs that were differentially expressed after exposure to ionizing radiation. miR-151-3p and miR-128-3p were identified as dose-specific markers of exposure to 8 Gy radiation [43]. A meta-analysis conducted by Małachowska et al. revealed that numerous studies employing varying doses of radiation consistently identified seven miRNAs (miR-150, miR-30a, miR-30c, miR-34a, miR-200b, miR-29a, miR-29b) whose expression was demonstrably altered in the aftermath of radiation exposure. Of these, four miRNAs (miR-150, miR-200b, miR-30c, miR-320a) demonstrated significant potential as biomarkers of radiation exposure, exhibiting a correlation with both exposure time and radiation dose [44].

However, the present study offers a unique contribution to this field in several ways. The majority of studies investigating miRNAs as biomarkers have been conducted in cell culture and animal models under a range of radiation exposure conditions. However, there is a paucity of research focusing on occupational exposure. It is important to note that data obtained from cell or animal culture may not be sufficient for the creation of a diagnostic tool for clinical use, which underscores the significance of our study. By directly analyzing the expression patterns of miRNAs in individuals exposed to X-ray radiation, it becomes possible to determine the precise miRNA signature that will facilitate the effective diagnosis of radiation exposure-related effects.

The results of our study indicate a decrease in the expression level of seven miRNAs (miR-19a-3p, miR-125b-5p, miR-142-5p, miR-144-5p, miR-150-5p, miR-181a-3p, miR-320c) and an increase in miR-29a-3p in the blood plasma of trauma surgeons who were exposed to X-rays as part of their professional activities. Our data on the expression profile of the studied miRNAs are consistent with the results of other studies. Song et al. demonstrated that the expression level of miR-144-5p was reduced in clinical samples of non-small cell lung cancer (NSCLC), as well as in radiation-exposed A549 and H460 cell lines [45]. In a study conducted by Fendler et al., a reduction in the levels of miR-19a-3p and miR-150-5p was observed following irradiation in rhesus macaques (M. mulatta) [46]. Acharya et al. found a significant decrease in the expression of miR-142-5p and miR-150-5p 1 and 7 days after irradiation with a dose of 2 Gray [47].

A dose-dependent effect of the miR-19a-3p and miR-125b-5p profile changes was identified in trauma surgeons. Additionally, a positive correlation was observed between the miR-29a-3p profile and the length of employment of trauma surgeons.

Exposure to radiation, including X-rays, can cause cell damage primarily through oxidative stress and the disruption of cellular metabolism. Mitochondria play a crucial role in the cell’s response to radiation by providing the necessary energy resources. During exposure to stress factors, mitochondria tend to maintain their structure and quantity in the cell. Mitochondria frequently alter their shape in response to physiological and pathological conditions [48]. Exposure to radiation results in the formation of significant amounts of ROS, which can damage mtDNA and mitochondrial proteins [49]. Low doses of radiation, defined as less than 0.1 Gy, lead to an increase in antioxidant defense and mitochondrial fission and fusion. On the other hand, high doses of radiation, defined as greater than 1 Gy, cause severe structural damage to cells. The cellular response to high doses of radiation depends on the integrity and functional stability of mitochondria [50].

Inhibition of mitochondrial functional activity is associated with disruption of respiratory complexes I (NADH dehydrogenase) and III (cytochrome c reductase) [51]. Barjaktarovic et al. reported a decrease in the activity of complexes I and III after X-ray irradiation with a dose of 2 Gy after 4 weeks [49]. The irradiation of mitochondria results in the loss of membrane potential, increased membrane permeability and subsequent release of cytochrome C. This deformation of mitochondrial membranes and disruption of their integrity initiates radiation-induced apoptosis [52]. As a result of apoptosis or necrosis, mtDNA fragments and circulates freely in the bloodstream, which allows the use of cf mtDNA as a potential biomarker of radiation exposure [53].

Thus, Borghini et al. showed that the level of circulating mtDNA in interventional cardiologists exposed to X-rays was two times higher than in the control group not exposed to occupational exposure [54].

Our previous study also showed a correlation between the level of cf mtDNA and the radon levels in residents of radon-prone regions in Kazakhstan who were exposed to alpha radiation [24].

In the present study, a similar trend of increased levels of cf mtDNA in the blood plasma was observed in individuals exposed to professional X-ray radiation. The level of cf mtDNA in the trauma surgeon group was more than three times higher than in the control group. This effect is specifically attributed to X-rays rather than confounding factors such as smoking. The combination of X-ray exposure and smoking was found to exert a synergistic effect. Presumably, the difference in the level of circulating mitochondrial DNA observed between the experimental and control groups of the study is associated with the destructive effects of X-ray on cells, leading to apoptotic cell death and the release of circulating mitochondrial DNA into the bloodstream. Moreover, radiation has the ability to enhance mitochondrial biogenesis and the expression of mitochondrial genes and subsequently increase the number of mitochondrial copies in order to generate more ATP [22,53]. 

It is important to note that the number of articles providing information about the role of cf mtDNA as a marker of radiation damage is currently limited. Our previous research [24] and the results of the current study convincingly demonstrate that the level of cf mtDNA is highly responsive to radiation exposure, regardless of the type of radiation.

miRNAs are capable of regulating the expression of multiple target genes, thereby exerting control over a multitude of cellular processes. Bioinformatics analysis revealed that miR-29a-3p is a target of VDAC1. VDAC1 is located on the outer mitochondrial membrane and is a multifunctional channel protein. As a mitochondrial gatekeeper, VDAC1 regulates numerous mitochondrial functions and also supports mitochondrial metabolism by facilitating the transport of substances (Ca^2+^, nucleotides, ATP, fatty acids and reactive oxygen species) from the cytosol and back [55]. The overexpression and further oligomerization of VDAC1 mediates the release of proapoptotic proteins and induces apoptosis. However, interaction with Bcl-2 and Bcl-xL leads to a decrease in apoptotic activity and cell survival [56]. A study by Skonieczna et al. demonstrated that the inhibition of VDAC led to increased ionizing-radiation-induced apoptosis and increased ROS synthesis [57]. Thus, it can be hypothesized that the overexpression of miR-29a-3p activates apoptosis by targeting VDAC1. The induction of apoptosis by oxidative stress or the inhibition of VDAC1 resulted in a significant increase in the number of mitochondrial DNA fragments in the blood of individuals exposed to radiation. Consequently, it is established that cf mtDNA binds to Toll-like receptor 9, resulting in the expression of pro-inflammatory cytokines and the development of aseptic inflammation [58]. 

The role of miR-29a-3p in regulating mitochondrial activity through targeting VDAC1 suggests that this miRNA belongs to the class of MitomiR. In their review, Rencelj et al. also identified miR-29a-3p as a MitomiR, due to its involvement in lipid β-oxidation through targeting Foxa2 [59].

Using MIENTURNET, we determined that miR-19a-3p and miR-125b-5p target ALOX5. ALOX5 catalyzes the peroxidation of polyunsaturated fatty acids (PUFAs), including arachidonic acid (AA). Arachidonic acid is a substrate for the synthesis of leukotrienes, prostaglandins and thromboxanes, which are powerful mediators of the inflammatory response [60]. Thus, ALOX5 is capable of inducing apoptosis by two mechanisms: disruption of the integrity of the cytoplasmic membrane through phospholipid peroxidation [61] and triggering an inflammatory response. Furthermore, recent studies have indicated that elevated levels of ALOX5 are associated with autophagy and ferroptosis in vitro and in vivo [62]. Our data showed a decrease in the expression of miR-19a-3p and miR-125b-5p in response to radiation. Presumably, the depletion of these miRNAs leads to the normal synthesis of ALOX5 and the initiation of inflammation in response to radiation exposure. A review of the literature revealed that miR-19a/b-3p plays a role in the inflammatory processes that occur during ischemia/reperfusion injury by targeting the SIRT1/FoxO3/SPHK1 axis [63]. The overexpression of miR-19a/b following spinal cord injury has been demonstrated to promote neuroinflammation via microglial activation [64]. However, multiple studies have demonstrated that miR-125b-5p acts as a negative regulator of inflammation. miR-125b-5p stimulated apoptosis and inhibited the inflammatory response by targeting DRAM2 [65]. miR-125b-5p was found to regulate the expression of inflammatory genes in human osteoarthritis chondrocytes, in a manner that involved the targeting of the TRAF6/MAPKs/NF-κB signaling pathway [66]. 

The miRNAs we studied, miR-29a-3p, miR-19a-3p and miR-125b-5p, regulate the levels of VDAC1 and ALOX5 in the cell. VDAC1 and ALOX5, in turn, are participants in many cellular processes that trigger the inflammatory response. It is worth noting that X-rays generally cause oxidative damage to cellular compartments and DNA. The resulting damage or cell death also stimulates the secretion of inflammatory mediators such as cytokines and chemokines [67]. Ionizing radiation initiates the activation of multilevel signaling cascades, which may result in either apoptosis or cell survival, for instance, the stimulation of the pivotal transcription factor nuclear factor kappa B (NF-kB) [68]. NF-kB plays a pivotal role in the regulation of inflammatory processes, as it controls the expression of pro-inflammatory cytokines and chemokines, including TNF-α, IL1, IL2 and IL6 [69]. The expression of IL-6, induced by the NF-kB pathway, has been observed in HeLa cells in response to X-ray irradiation [70]. Linard et al. reported an increase in the expression of IL-1β, TNF-α and IL-6 mRNA at 3 and 6 h after irradiation, but the expression of IL-6 and IL-8 increased only after 3 days. While the level of anti-inflammatory IL-10 was significantly lower on Day 3 [71]. As shown by the results of the study by Shan et al., low (0.075 Gy) and high (2 Gy) doses of ionizing radiation stimulated the secretion of IL-12 and IL-18 through activation of the Toll signaling pathway in macrophages. Moreover, a dose-dependent effect of the synthesis of IL-12 and IL-18 was demonstrated at doses from 0.05 to 4 Gy [72]. Kiang et al. studied changes in the concentration of circulating cytokines/chemokines depending on the power of radiation exposure. The spectrum of IL-1β, IL-6, IL-10, IL-15, IL-17A, IL-18 and TNF-α changed after different periods of exposure to different doses of ionizing radiation [73]. Han et al. identified a decrease in the level of interferon (IFN)-gamma 3 h after irradiation with gamma rays, while the levels of IL-4, IL-5 and IL-10 were increased [48]. The balance between pro-inflammatory and anti-inflammatory cytokines is very important and can shift for a long time after irradiation, maintaining or disrupting cell homeostasis [9]. The findings of our study are entirely consistent with the prevailing understanding of the effects of radiation exposure on the human body. An increase in the levels of pro-inflammatory cytokines, such as TNF-α and IL-6, and a decrease in anti-inflammatory cytokines, such as IL-4, indicates the presence of an active inflammatory process [74]. The results of the regression analysis revealed a statistically significant, positive correlation between the level of miR-29a-3p expression and the level of pro-inflammatory IL-6 (*p* = 0.0092). Our study revealed elevated levels of miR-29a-3p and IL-6 in the blood plasma of trauma surgeons. High levels of IL-6 indicate an ongoing inflammatory process in study participants. We observed a positive correlation between the level of IL-6 and the length of employment of trauma surgeons.

The multifaceted roles of miRNAs in various cellular processes are well documented. The same miRNAs can be involved in several processes that are in opposition to each other. Additionally, numerous signaling cascades are regulated by RNA molecules. The results of our study indicate that the miRNA profile and the level of cf mtDNA may serve as promising markers of radiation exposure. The levels of mtDNA, miR-19a-3p and miR-125b-5p demonstrated an association with the effective annual dose of radiation exposure. Consequently, the identification of radiosensitive and dose-specific biomarkers will facilitate the development of a panel of markers for the rapid diagnostic screening of individuals at risk of developing radiation syndromes.

## 4. Materials and Methods

### 4.1. Subjects

The experimental group included 30 trauma surgeons who were regularly exposed to X-ray radiation. The blood was collected at the Research Institute of Traumatology and Orthopedics named after the academician N.D. Batpenov (Astana, Kazakhstan).

The following criteria were used to include participants in the experimental group: healthy individuals without chronic and inflammatory diseases, living in an area with low radiation exposure (including radon) and with work experience of at least one year.

The X-ray radiation dose was monitored using a thermoluminescent dosimeter (TLD-3). All trauma surgeons who were included in the experimental group were provided with the aforementioned device.

The control group included 56 healthy donors. The collection was carried out at the Research and Production Center for Transfusiology in Astana. A laboratory blood test confirmed the absence of inflammatory diseases. In order to be included in the control group, participants were required to meet a number of criteria. These included the absence of chronic inflammatory diseases, a profession not associated with sources of ionizing radiation, residence in an area with a low level of radiation pollution (including radon) over the past five years and the absence of medical manipulations associated with exposure to ionizing radiation. 

All data were collected via a survey process. Voluntary consent was obtained from all study participants.

The study was approved by the Ethics Committee of the Medical University of Astana (Protocol No. 4). Voluntary consent was obtained from all participants in the study.

### 4.2. Calculation of Individual Annual Effective Doses for Trauma Surgeons

Dosimetric monitoring for personnel is carried out quarterly based on the readings of individual dosimeters (thermoluminescent dosimeter—TLD-3). The values corresponding to the dosimeter readings are taken as the value of the quarterly effective dose. The doses created by the natural background are also measured. During the exposure of working dosimeters, background dosimeters are stored on the territory of the institution in a room remote from any sources of radiation. Dose values from the natural background are subtracted from the readings of exposed individual dosimeters. The individual annual effective radiation dose is equal to the sums of the corresponding individual doses based on the results of quarterly monitoring during the calendar year.

To read the data, the dosimeters were connected to the DVG-02TM installation («DOZA» Research and Production Enterprise), which provides individual dosimetric monitoring.

### 4.3. Preparation of Blood Samples

Blood samples were collected into hematology vacuum tubes filled with EDTA K3 and clotting activator gel. Samples were centrifuged to separate plasma no more than 4 h after blood collection (10 min, 1500 g). Samples were stored at −80 °C.

### 4.4. miRNA Extraction

Total RNA including miRNAs was isolated from blood plasma using the commercial miRNeasy Serum/Plasma Advanced Kit (50) (#217204, Qiagen, Germany) according to the manufacturer’s protocol. The concentration and purity of the isolated RNA fraction was measured using a NanoDrop™ One Microvolume UV–Vis Spectrophotometer (#ND-2000, ThermoFisher Scientific, Inc., Waltham, MA, USA).

### 4.5. PCR Analysis of miRNA Expression

The expression of selected miRNAs was determined using RT-PCR on an Applied Biosystems QuantStudio™ 3 cycler. cDNA was synthesized using a commercial reverse transcription kit miRCURY LNA RT Kit (#339340, Qiagen, Germany) according to the manufacturer’s protocol. Each PCR reaction included miRCURY LNA SYBR^®®^ Green PCR Kits (#339347, Qiagen, Germany), specific primers and cDNAs. The PCR program consisted of the following steps: 95 °C for 10 min and 40 cycles (95 °C for 15 s and 56 °C for 1 min). All samples were amplified in two runs. U6 was used as the endogenous reference gene. The 2^−ΔΔCt^ method was used to determine the level of miRNA expression [75,76].

### 4.6. DNA Extraction

The extraction of total extracellular DNA from blood plasma samples was performed using the “PROBA-NK/PROBA-NK-PLUS” reagent kit (#D07-2, DNA-Technology, Moscow, Russia) following the manufacturer’s protocol. The measurement of extracellular DNA concentration in the samples was performed using a Nanodrop 1000 spectrophotometer (Thermo Fisher Scientific, Waltham, MA, USA).

### 4.7. PCR Analysis

The determination of the level of cf mtDNA was performed using qPCR. All samples were analyzed in duplicate using the QuantStudio™ 3 Real-Time PCR System (Thermo Fisher Scientific, Waltham, MA, USA).

As a target for the detection and quantification of cf mtDNA, the mt-specific 16S rRNA (230 bp) was selected. The primer sequence and PCR program are presented in Table 6. The primers were synthesized by Syntol LLC (#2774, Moscow, Russia).

The reaction mixture, with a total volume of 25 μL, consisted of 12.5 μL of Thermo Scientific Maxima SYBR Green/ROX qPCR Master Mix (2X) (#K0222, Thermo Fisher Scientific, Waltham, MA, USA), 20 pmol of forward/reverse primers and 100 ng of DNA sample and was brought to the final volume with nuclease-free deionized water.

### 4.8. Detection of Cytokines in Serum

The serum levels of TNF-α, IL-6 and IL-4 were determined by ELISA using the commercial set of reagents “Vector-Best” (Novosibirsk, Russia) according to the manufacturer’s protocol. The optical density of each well was read at 450 nm using an Immunochem-2100 microplate reader (High Technology Inc., Attleborough, MA, USA). Cytokine concentrations in each serum sample were calculated using standard curves of known cytokine concentrations.

### 4.9. Statistical Analysis

Statistical data analysis was performed using GraphPad Prism 9 software (GraphPad Software, Inc., La Jolla, CA, USA). Data were presented as the mean ± standard deviation (SD). Differences between the experimental and control groups were assessed using the *t* test (for Student’s t distribution) or the Mann–Whitney U test (for non-normal distribution). Correlation analysis was carried out using the Pearson or Spearman coefficient. Multiple linear regression was used to assess the impact of age, gender and smoking on changes in the miRNAs profile and cf mtDNA level. *p* < 0.05 was considered statistically significant (* *p* < 0.05; ** *p* < 0.01; *** *p* < 0.001; **** *p* < 0.0001). ROC curves analysis was performed using MedCalc Software version 22.026 (Belgium, Bruxelles).

### 4.10. Target Enrichment Analysis

The miRNA enrichment turned network (MIENTURNET, Rome, Italy) was used to construct interaction networks between miRNAs and their targets. We uploaded into the MIENTURNET web tool (http://userver.bio.uniroma1.it/apps/mienturnet/, accessed on 1 May 2024) a list of miRNAs whose profile changed under the influence of X-rays, which was confirmed by qRT-PCR. miRNA–target interaction networks were created using the miRTarBase database. To obtain more stringent criteria, we set the following MIENTURNET parameters: threshold for the minimum number of miRNA–target interactions—2, threshold for the adjusted *p*-value (FDR) = 0.05 and filter by the category of evidence “Strong”.

## 5. Conclusions

Ionizing radiation represents one of the most significant environmental factors affecting human health, with the potential to lead to the development of a range of diseases, including cancer.

Currently, cytogenetic analysis is employed as a means of identifying biomarkers of radiation exposure. However, this approach is not always readily available, convenient, or time-efficient. Epigenetic and molecular changes could prove to be more useful and sensitive biomarkers. Liquid biopsy represents one of the most promising areas of modern diagnostics, characterized by its ease of use.

miRNAs, which have been demonstrated to be effective biomarkers for a range of diseases, can also be employed to assess the impact of ionizing radiation on humans.

This is particularly pertinent in the case of MitomiRs, which regulate the expression of mitochondrial genes. Mitochondria play a pivotal role in the cellular response to radiation exposure. Mitochondrial dysfunction caused by radiation exposure leads to cell death and, as a consequence, an increase in the level of cf mtDNA.

Our study identified promising miRNAs that showed a dose-dependent effect of X-ray exposure, a correlation with inflammatory cytokines and, according to bioinformatics analysis, target proteins involved in mitochondrial transport and the inflammatory response.

In conclusion, a series of differentially expressed miRNAs induced by X-ray has been identified, including miR-19a-3p, miR-125b-5p, miR-142-5p, miR-144-5p, miR-150-5p, miR-181a-3p, miR-320c and miR-29a-3p. The findings indicate the potential for the use of miRNA profiling and cf mtDNA levels as non-invasive biomarkers for the assessment of X-ray exposure risk.

## Figures and Tables

**Table 1:** Comparative characteristics of the study participants.

Characteristic		Control *n* = 56 (%)	Trauma Surgeons *n* = 30 (%)
**Gender**	Male	25 (45)	19 (63)
	Female	31 (55)	11 (27)
Age (years)	≥40	15 (27)	10 (33)
	<40	41 (73)	20 (67)
Smoking	Nonsmoker	21 (38)	24 (80)
	Former smoker	2 (2)	3 (10)
	Current smoker	33 (60)	3 (10)
Work experience	1–5 years		13 (43)
	6–10 years		4 (16)
	>10 years		13 (43)
Number of working hours per day	1–5 h		11 (37)
	6–8 h		11 (37)
	>8 h		8 (26)

**Table 2:** Values of individual annual effective doses for trauma surgeons.

Sample	AED (mSv)	Sample	AED (mSv)	Sample	AED (mSv)	Sample	AED (mSv)	Sample	AED (mSv)
1T	2.2	7T	0.2	13T	0.26	19T	0.72	25T	0.28
2T	2.26	8T	0.22	14T	0.28	20T	0.69	26T	0.26
3T	0.81	9T	0.23	15T	0.29	21T	0.79	27T	0.2
4T	0.82	10T	0.24	16T	0.32	22T	0.29	28T	0.82
5T	0.81	11T	0.24	17T	0.24	23T	0.29	29T	0.32
6T	1.98	12T	0.25	18T	0.71	24T	0.32	30T	0.72

**Table 3:** Copy numbers of the cf mtDNA.

Parameter	Control, Copies/mL	Trauma Surgeons, Copies/mL	*p* Value
Median in both smokers and non-smokers	1.21 × 10^5^	5.28 × 10^5^	0.0005
Median nonsmokers	1.03 × 10^5^	3.18 × 10^5^	0.01
Median smokers	1.45 × 10^5^	1.38 × 10^7^	0.003

**Table 4:** Multivariate linear regression: the impact of age, gender and smoking on changes in the miRNAs profile and cf mtDNA level.

	F (DFn.DFd); *p* Value		
	Age	Gender	Smoking
miR-19a-3p	F (1.80) = 1.229 *p* = 0.2709	F (1.80) = 0.5105 *p* = 0.4770	F (2.80) = 0.2510 *p* = 0.7787
miR-29a-3p	F (1.54) = 0.1488 *p* = 0.7012	F (1.54) = 2.348 *p* = 0.1313	F (2.54) = 0.7915 *p* = 0.4584
miR-125b-5p	F (1.61) = 0.07672 *p* = 0.7827	F (1.61) = 0.3474 *p* = 0.5577	F (2.61) = 1.457 *p* = 0.2409
miR-142-5p	F (1.81) = 0.4522 *p* = 0.5032	F (1.81) = 0.9459 *p* = 0.3337	F (2.81) = 3.330 *p* = 0.0407
miR-144-5p	F (1.54) = 0.03664 *p* = 0.8489	F (1.54) = 3.254 *p* = 0.0768	F (2.54) = 0.5587 *p* = 0.5752
miR-150-5p	F (1.77) = 2.176 *p* = 0.1442	F (1.77) = 0.08428 *p* = 0.7724	F (2.77) = 2.578 *p* = 0.0825
miR-181a-3p	F (1.75) = 0.1840 *p* = 0.6692	F (1.75) = 0.1485 *p* = 0.7011	F (2.75) = 0.3031 *p* = 0.7394
miR-320c	F (1.59) = 0.2414 *p* = 0.6250	F (1.59) = 1.471 *p* = 0.2301	F (2.59) = 1.304 *p* = 0.2790
cf mtDNA	F (1.80) = 2.562 *p* = 0.1134	F (1.80) = 0.2057 *p* = 0.6514	-

**Table 5:** Assessment of the potential of miRNAs and cf mtDNA as biomarkers of X-ray exposure.

Area under the ROC Curve (AUC)	miR-19a- 3p	miR-29a- 3p	miR-125b-5p	miR-142- 5p	miR-144- 5p	miR-150- 5p	miR-181a-3p	miR-320c	cf mtDNA
95% Confidence interval	0.615 to 0.867	0.674 to 0.907	0.604 to 0.859	0.852 to 0.992	0.650 to 0.891	0.599 to 0.855	0.717 to 0.933	0.737 to 0.944	0.700 to 0.923
Significance level *p*	0.0009	<0.0001	0.0018	<0.0001	<0.0001	0.0019	<0.0001	<0.0001	<0.0001
Sensitivity	76.19	80.95	85.71	85.71	80.95	80.95	80.95	85.71	100
Specificity	82.76	82.76	79.31	96.55	75.86	72.41	86.21	82.76	62.07

**Table 6:** qPCR protocol.

Group	Primers	Nucleotide Sequence (5’−3’)	qPCR Program
Population	forward	5’-CAGCCGCTATTAAAGGTTCG-3’	90 °C—10 min; (95 °C—15 s, 60 °C—60 s) × 40
	reverse	5’-GGGCTCTGCCATCTTAACAA-3’	

## Data Availability

Data are contained within the article.
